# Predicting the Engineering Properties of Rocks from Textural Characteristics Using Some Soft Computing Approaches

**DOI:** 10.3390/ma15227922

**Published:** 2022-11-09

**Authors:** Davood Fereidooni, Luís Sousa

**Affiliations:** 1 School of Earth Sciences, Damghan University, Damghan 36716-41167, Iran; 2 Department of Geology and Pole of CGeo—Geoscience Center, University of Trás-os-Montes e Alto Douro, 5000-801 Vila Real, Portugal

**Keywords:** rock, engineering properties, texture coefficient, statistical method, artificial neural network

## Abstract

Rock is used as a foundation and building material in many engineering projects and it is important to determine/predict its engineering properties before project construction. Petrographic and textural characteristics are useful parameters for predicting engineering properties of rocks in such applications. In this research, fifteen rock samples were taken and their engineering characteristics, namely dry and saturated unit weights, porosity, water absorption, slake durability index (SDI), Schmidt rebound hardness (SRH), ultrasonic P-wave velocity (UPV), and uniaxial compressive strength (UCS), were measured in the laboratory. Petrographic and textural characteristics of the rocks, determined from thin section and X-ray diffraction investigations, led to the evaluation of the texture coefficient (TC). Based on simple regression analysis (SRA), the TC values have direct relationships with density, SDI, SRH, UPV, and UCS, and inverse relationships with porosity and water absorption. Experimental models were developed using multiple regression analysis (MRA) and artificial neural network (ANN) to predict Id_2_, SRH, UPV, and UCS of the tested rocks from the values of TC. Some statistical parameters including Pearson regression coefficient (R), coefficient values account for (VAF), root mean square error (RMSE), mean absolute percentage error (MAPE), and performance index (PI) were calculated to assess the performances of the MRA and ANN models. The correlations between experimental and calculated values of Id_2_, SRH, UPV, and UCS indicated that predicted values of the ANN models are more valid than the MRA. Additionally, the residual error of the ANN models varies less than the MRA. Finally, it has been concluded that the SRA, MRA, and ANN methods can successfully predict the rock engineering properties from the TC.

## 1. Introduction

Natural stones are used worldwide at an increasing rate because of their availability, excellent physical-mechanical properties, and diversity of textural characteristics [1,2]. Several factors are considered during an exploration campaign of building stones, such as the fracturing in the quarry, which defines if a rock mass can be exploited, or the durability, which delimits the possible applications [3,4,5,6,7,8]. The evaluation of the geotechnical properties of building stones, such as color and brightness, is a key factor to define their utilization [9,10,11,12,13,14]. Furthermore, engineering characteristics of different rocks are essential parameters for design and construction of various engineering projects such as dams, tunnels, caverns, and foundations either on or inside rocks. These characteristics could be determined practically through direct methods as described in ISRM [15]. Applying direct tests to assess these properties is usually expensive, time consuming, and requires regularly shaped samples. Therefore, in such cases, experimental approaches and empirical equations can be used to predict the engineering properties of rocks from simple and index test results.

Several researchers have attempted to develop various soft computing models for predicting different parameters from others in material sciences [16,17,18,19,20,21,22,23,24,25] and engineering properties of different rock types from their petrographic characteristics in engineering geology and rock mechanics [26,27,28,29,30,31,32,33,34,35]. In the recent years, some research works have been performed to assess correlations between mineralogical and textural characteristics and mechanical properties of different rocks by using statistical analyses and different soft computing approaches such as genetic programing (GP), artificial neural network (ANN), adaptive neuro-fuzzy inference system (ANFIS), and support vector machine (SVM) [36,37,38,39,40,41,42,43,44,45,46,47,48,49,50,51,52]. The main advantages of these approaches are that (i) they have made it possible to solve nonlinear problems, in which mathematical models are not available, and (ii) they have introduced human knowledge such as cognition, recognition, understanding, learning, and others in the fields of computing [53]. In this regard, Brace [54] recognized that fine-grained rocks have higher strength than coarse-grained ones. Ulusay et al. [55] tested sandstones in a laboratory and found that textural properties are important parameters for predicting engineering properties of these rocks. Tugrul and Zarif [56] stated that textural characteristics are more important than mineral content for estimating the engineering properties of rocks. Eberli et al. [57] observed that the size and shape of grains, grain size distribution, and the ratio of grain to matrix have an influence on the acoustic wave velocity through the unconsolidated carbonate sediments. Meng and Pan [58] and Khanlari et al. [59] indicated that physical properties (i.e., density, porosity, permeability) and mechanical characteristics (such as strength, deformability, durability, and hardness) of rocks are affected by mineral composition, grain size, grain contact, and rock cement. Jensen et al. [38] found that porosity, grain size, weakness planes, and microfractures presented in limestones’ texture have important effects on the strength of the rocks.

The texture coefficient (TC) was developed by Howarth and Rowlands [60] to understand the effect of textural characteristics on the physical and mechanical properties of rocks. They investigated the relations between the mechanical properties and TC for igneous rocks, marbles, and sandstones and found close relationships between mechanical rock properties and the TC. In many research studies, the TC has been applied to predict the engineering properties of rocks [61,62,63,64]. Singh et al. [36] used artificial neural networks to estimate the strength of schistose rocks from their petrographic characteristics. In their research, the network was trained to predict axial point load, uniaxial compression, and tensile strengths from mineralogical content, grain size, aspect ratio, surface weight, form factor, and foliation orientation. Alber and Kahraman [65] used regression analysis to predict the elasticity modulus and uniaxial compressive strength of a fault breccia from the TC. The results of their research remarked that the uniaxial compressive strength of the tested breccia could be estimated from the texture coefficient. Manouchehrian et al. [42] applied artificial neural networks and multivariate regression for assessing the uniaxial compressive strength based on textural characteristics of rocks. Ersoy and Acar [66] studied the petrographic and textural properties’ influences on the strength of granites and found that the size of mineral has a higher effect than mineral type on rock strength. Aligholi et al. [67] evaluated relationships between drillability indices on one hand and index properties and petrographic data on the other hand of hard igneous rocks. They revealed that the multiple regression models prepared by using petrographic features provide a better prediction of drillability in comparison to those prepared by using index properties. Akram et al. [68], by assessing petrographic and mechanical characteristics of Sakesar limestone, attempted to establish the relationships between the parameters and the strength parameters such as uniaxial compressive strength, point load index, and Schmidt rebound hardness values. In their research, regression analyses were used to establish correlations between limestone constituents and the parameters of strength. Kolay and Baser [69] focused on the relationships between internal structure and engineering parameters of basalt rocks by digitizing the textural properties. In this research, simple regression analysis was performed using the laboratory results incorporating texture coefficient and engineering parameters including dry unit weight, P-wave velocity, Schmidt rebound hardness, point load strength index, and uniaxial compressive strength of the rocks. Their results showed that the strength of the basalts has direct relation by their texture coefficient.

All the above studies show that the petrographic characteristics and rock texture affect the engineering properties of different rocks. These properties can be easily assessed by routine thin section studies in a laboratory. As sedimentary rocks have a very diverse nature and suitable texture in the presence of grains with different sizes and shapes in microscopic studies, the effect of petrographic and textural characteristics on engineering properties of sedimentary rocks is sensible. Therefore, this research focuses on the assessment of engineering properties and the textural coefficient of the rocks to predict the engineering properties from petrographic and textural characteristics using statistical methods and artificial neural networks. So, the main objective of the research is to establish relationships between the textural characteristics of rocks as a primary and essential parameter from one side and engineering properties as advanced and perfect parameters from the other by using careful approaches such as soft computing methods. Additionally, another benefit of this research is that the use of soft computing methods for predicting rock engineering characteristics is a cheap and quick method, especially for soft rocks such as sedimentary rocks, for which laboratory tests are difficult. On the other hand, this research can be considered as a model for future research and its results can be used by other researchers. These issues motivated this research. Therefore, fifteen sedimentary rock samples including limestones and sandstones have been studied to predict their engineering properties from petrographic and textural characteristics using statistical analysis and soft computing methods.

## 2. Geological Setting and Methodology

**Figure 1:**
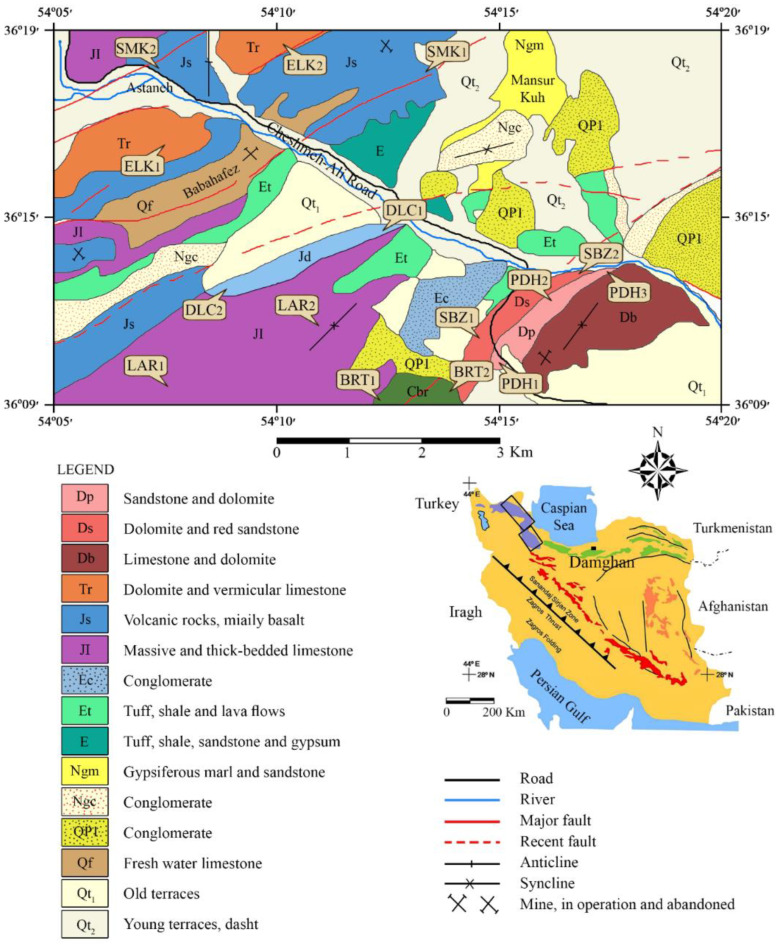
Sampling locations and geological map of the study area [70].

The study area and sampling locations are placed in the north and northwest of the Damghan region in the north of Iran. This region has a continental climate and an irregular topography related to its geological history. The area is situated in the Alborz-Azarbayejan structural zone and contains Paleozoic to Neogene outcropped rocks. Suitable sampling locations were found from the 1:100,000 geological map presented by the Geological Society of Iran [70]. Fifteen rock samples including limestones and sandstones were collected for performing the study. Sampling was carried out from quarries, trenches, and road cuttings during field investigations. The limestone samples include Sibzar (SBZ), Elika (ELK), Dalichai (DLC), and Lar (LAR) and the sandstone samples contain Padeha (PDH), Barut (BRT), and Shemshak (SMK). SBZ samples from the Middle Devonian age were collected from the Bahram and Sibzar formations from the northern part of a ballast mine in the region. ELK samples from the Lower Triassic age were collected from the Elika formation from a new trench near the Astaneh village. DLC and LAR samples, which depend on the Dalichai and Lar formations of the Upper Jurassic age were collected from road cuttings. The samples of PDH were obtained from a ballast mine 10 km from the Chesmeh-Ali road. The age of the Padeha formation is Devonian. The samples of BRT were obtained from the Barut formation with a Lower Cambrian age. The samples of SMK were collected from the Shemshak formation outcropped near a travertine quarry. These samples are from the Middle Jurassic age. Figure 1 illustrates the geological properties and the sampling locations.

**Figure 2:**
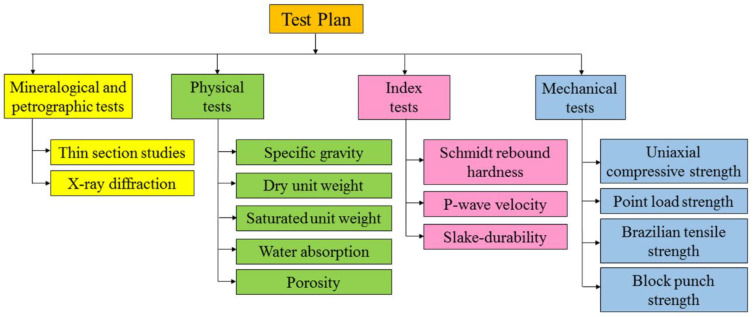
Test plan in this study for determining different properties of the rocks.

**Figure 3:**
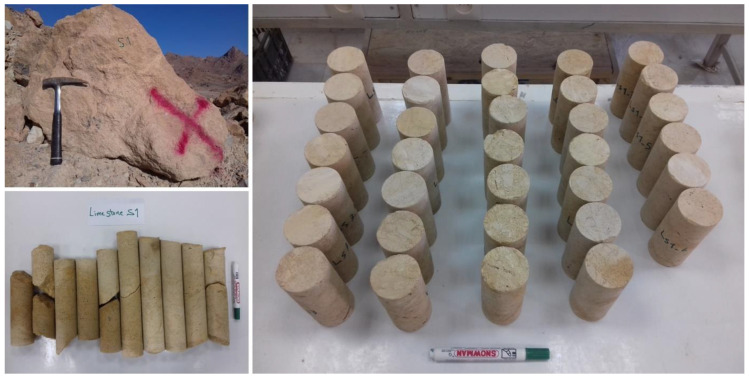
Block samples in the field and core specimens of the rocks.

**Figure 4:**
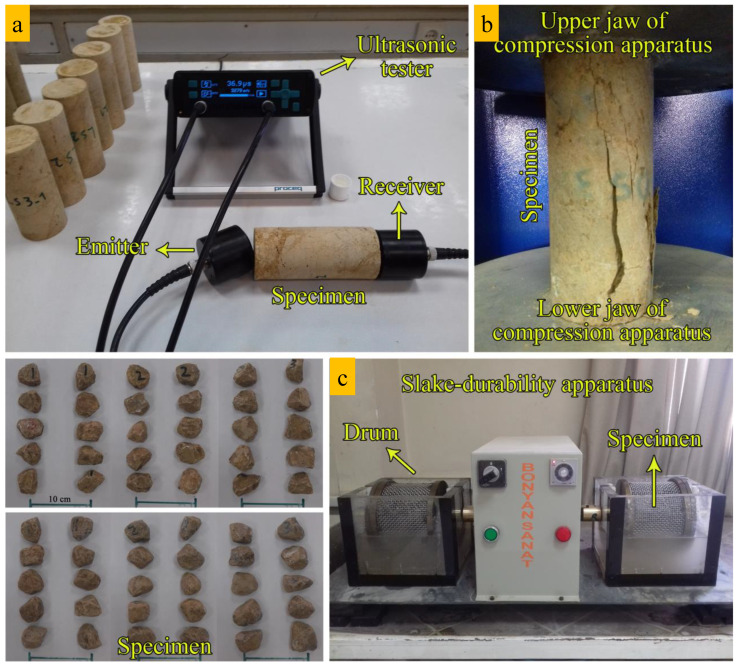
Testing procedure and the used apparatuses for the study. (**a**) P-wave velocity test, (**b**) uniaxial compressive test, and (**c**) slake durability test.

After the field investigations, a regular laboratory test program was carried out to assess engineering properties of the collected rocks. XRD analyses and microscopic investigations were performed based on ASTM [71] and ISRM [15] to determine the rocks’ constituent minerals, petrography properties, textural characteristics, and finally texture coefficient (TC). X-ray diffraction (XRD) analysis was carried out by X’Pert HighScore plus (v.3.0.5) software. Composition materials of the studied rocks were identified in the 2θ ranges from 4° to 70°. The dry unit weight, saturated unit weight, effective porosity, water absorption, P-wave velocity, Schmidt hardness, slake durability index, and uniaxial compressive strength of the rocks were measured. The test plan for the present study is summarized in Figure 2. These tests were conducted on standard NX core specimens (prepared based on ISRM [15]) presented in Figure 3. Additionally, the used apparatuses and testing procedures in this study are shown in Figure 4. Physical properties including dry and saturated unit weights (γ_dry_, γ_sat_), effective porosity (n), and water absorption (W_a_) were determined in accordance with ISRM [15]. The slake durability index (SDI) test was carried out in accordance with ISRM [15] and ASTM [72] up to 3 cycles. This test was performed on 150 irregular or lump prepared specimens. The Schmidt rebound hardness (SRH) test was carried out according to ISRM [73] and ASTM [74] on blocks of the rock samples. The ultrasonic P-wave velocity (UPV) test was conducted based on ASTM [75] in the laboratory. The uniaxial compressive strength test was conducted in accordance with ISRM [15] and ASTM [76]. Five experiments for each rock sample were performed on cylindrical specimens with a length to diameter ratio of 2:2.5 to determine the average values of UCS. Finally, the estimation of engineering properties of the rocks was performed from petrographic and textural characteristics using statistical methods and artificial neural networks.

## 3. Results of the Laboratory Investigations

### 3.1. Mineralogical and Petrographic Studies

**Figure 5:**
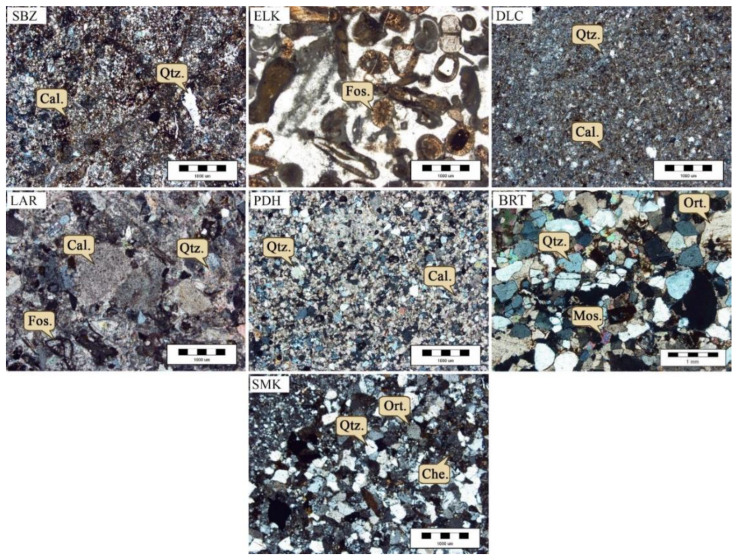
Microscopic images of the studied rock samples. (Qtz.: Quartz (SiO_2_); Cal.: Calcite (CaCO_3_); Dol.: Dolomite (Ca.Mg(CO_3_)_2_); Fld.: Feldspar; Fos.: Fossil).

Based on the obtained results, the studied limestones are generally composed of calcite, and the studied sandstones are quartz-rich (see Table 1 and Figure 5). The SBZ samples are sparry limestone (type I) or dolomitic limestone. These samples mainly contain calcite, dolomite, and quartz. The original calcite matrix has been replaced by dolomite, but the micritic allochems are partly replaced. Where replacement is incomplete, euhedral rhombe-shaped crystals are visible. ELK samples are mainly composed of ooids, peloids, and many fossils such as alga and echinoderms. The allochems are a mixture of ooids and bioclasts. Therefore, these samples are oosparite (type I) according to the Folk or ooid grainstone according to the Dunham classifications. Since the allochems are rounded, it would be a rounded oosparite using the Folk textural spectrum. DLC samples are sparry allochemical limestone (type I) or sandy grainstone. LAR samples are microcrystalline limestone (type III) without allochems. These samples are mainly composed of ooids, peloids, alga, and echinoderms. According to the Folk classification, PDH samples are typical greywacke that are dominantly composed of calcite, quartz, and dolomite. Poor sorting, abundant quartz monocrystalline, sedimentary origin, and the existence of rhombohedra dolomite are the main textural characteristics of these samples. Additionally, the rock matrix is composed of a hematite and dolomite fill between the fine-grain quartzes in these samples. BRT samples are litharenite and are composed of quartz, calcite, orthoclase, muscovite and others rock fragments such as chert. Quartz is the main mineral in these samples and calcite and feldspar are accessory minerals. SMK samples are classified as sub-litharenite and are composed of quartz (more than 70%) as the main mineral, and calcite and feldspar as accessory minerals. In these samples, the matrix fill is calcite and hematite between quartzes. The mineralogical and petrographic properties of the studied rocks are summarized in Table 1.

### 3.2. XRD Analysis

**Figure 6:**
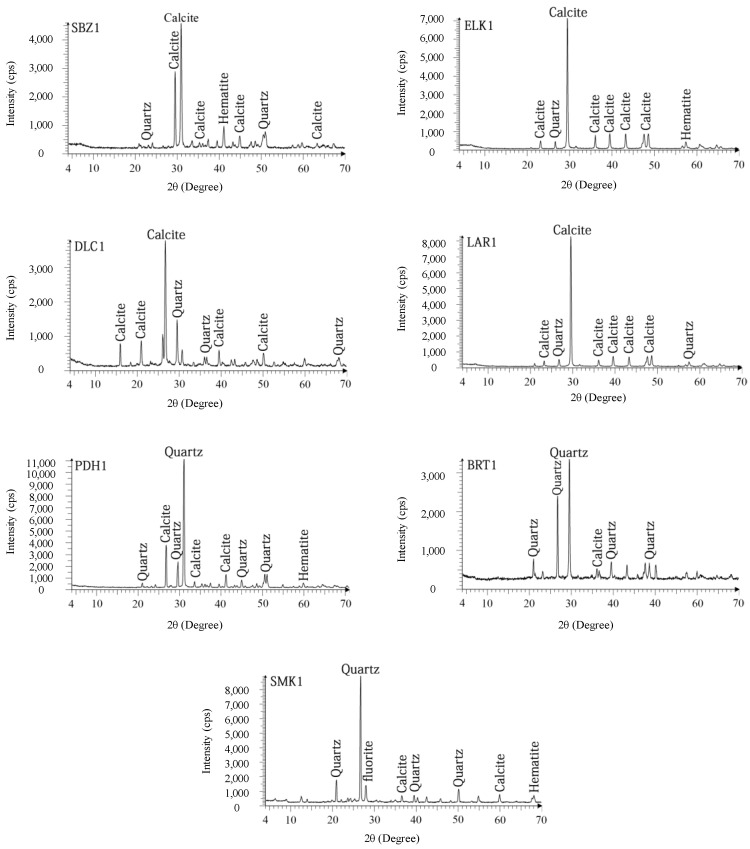
XRD graphs of the studied rock samples.

X-ray diffraction (XRD) analysis was performed and the provided diffractograms are shown in Figure 6. The results indicated that calcite and quartz are the two main minerals in all studied rocks. Other minerals such as hematite are present as secondary minerals in the rock samples. Similar to the thin section results, the limestones and sandstones are commonly composed of calcite and quartz, respectively. So, the results of both thin section studies and XRD analysis are completely coordinated with each other.

### 3.3. Engineering Properties Evaluation

The values of all physical properties of the studied rock samples including dry and saturated unit weights (γ_dry_, γ_sat_), effective porosity (n), and water absorption (W_a_) are presented in Table 2. The values of γ_dry_ are between 2.35 and 2.60 for the samples of PDH_3_ and SMK_2,_ respectively. Additionally, the values of porosity are between 2 and 10.32 for the samples of SMK_2_ and PDH_3_, respectively. Based on the IAEG [77] rock classification, most of the samples were placed in the rock groups with moderate to high unit weight and low to medium porosity. The samples of PDH_3_ and SMK_2_ have the lowest and highest values of the slake durability index equal to 95.54% and 99.21% at the third cycle (Id_3_), respectively (Table 2). According to the Gamble [78] classification, the tested samples were placed in durable and very durable rock groups. The lowest and highest values of the Schmidt rebound hardness (SRH) are between 24 and 49, which belong to the samples of PDH_3_ and SMK_2_, respectively. The value of ultrasonic P-wave velocity (UPV) ranges from 3.09 to 5.75 km/s. The samples PDH_3_ and LAR_2_ have the highest and lowest values of wave velocities, respectively. The minimum and maximum values of the uniaxial compressive strength (UCS) are between 16.09 and 127.43 MPa, which belong to the samples of PDH_3_ and SMK_2_, respectively. According to Broch and Franklin [79], the tested samples were placed in weak to extremely strong rock groups. The values of engineering properties for the studied rock samples are presented in Table 2.

Some comprehensive linear correlations were obtained between the physical and mechanical properties, which are presented in Table 3. Regression coefficients (R) as Pearson correlations were found between 0.77 and 0.98. Significant level values (Sig.) were between 0.000 and 0.001, and the high values of R show that the determined engineering parameters are significantly related to each other. Based on the results, Gs, γ_dry_, γ_sat_, SDI, SRH, UPV, and UCS have direct linear relations, whereas n and W_a_ have inverse linear relations to the other parameters. These results are comparable to the results presented by other authors [34,67,69,80].

### 3.4. Texture Coefficient (TC) Calculation

The texture of rocks was defined as “the degree of crystallinity, grain size or granularity, and the fabric or geometrical relationships between the constituents of a rock” [81]. The texture coefficient (TC) proposed by Howard and Rowlands [60] is a very good factor to quantify rock texture. The values of TC, which is the so-called rock fabric coefficient by Prikryl [82], were evaluated by performing four processes, namely (1) measuring grain circularity, (2) measuring grain elongation, (3) measuring and quantifying grain orientation, and (4) weighting of results based on the degree of grain packing [61]. After passing the processes, the TC is calculated from the following equation [61]:

(1) $$\mathrm{TC}=\mathrm{AW}\left[\left(\frac{N_{0}}{N_{0}+N_{1}}\times \frac{1}{{\mathrm{FF}}_{0}}\right)+\left(\frac{N_{1}}{N_{0}+N_{1}}\times {\mathrm{AR}}_{1}\times {\mathrm{AF}}_{1}\right)\right]$$

Here, TC is the texture coefficient, AW is the grain packing weighting, N_0_ is the number of grains whose aspect ratio is below a pre-set discrimination level, N_1_ is the number of grains whose aspect ratio is above a pre-set discrimination level, FF_0_ is the arithmetic mean of discriminated form factors, AR_1_ is the arithmetic mean of discriminated aspect ratios, and AF_1_ is the angle factor, quantifying grain orientation.

The AW, AR, and FF are calculated by the following equations [61]:

(2) $$\mathrm{AW}=\;\sum \frac{\left(\mathrm{grain}\;\mathrm{areas}\;\mathrm{within}\;\mathrm{the}\;\mathrm{reference}\;\mathrm{area}\;\mathrm{boundary}\right)}{\left(\mathrm{area}\;\mathrm{boundary}\;\mathrm{by}\;\mathrm{the}\;\mathrm{reference}\;\mathrm{area}\;\mathrm{boundary}\right)}$$

(3) $$\mathrm{AR}=\frac{L}{W}$$

(4) $$\mathrm{FF}=\frac{4\pi A}{P^{2}}$$

In these equations, L is length, W is width, P is the perimeter, and A is the area. The angle factor (AF) is calculated by the equations below [61]:

(5) $$\mathrm{AF}=\sum_{i=1}^{9}\left(\frac{x_{i}}{\frac{N\left(N-1\right)}{2}}\right)\times i$$

(6) $${\mathrm{AF}}_{1}=\frac{\mathrm{AF}}{5}$$

where x_i_ is the number of angular differences in each class, N is the total number of elongated grains, and i is the weighting factor and class number.

**Figure 7:**
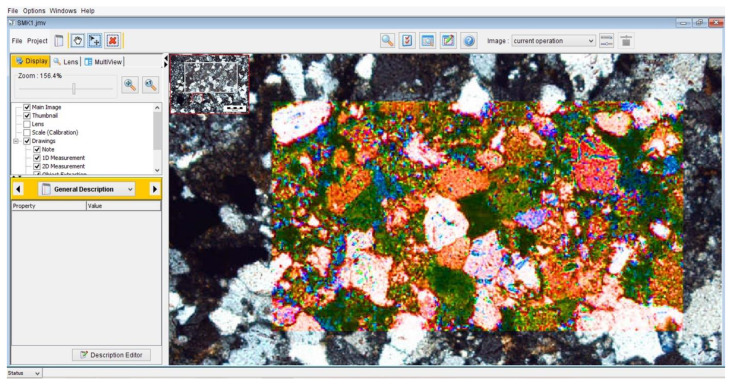
JMicroVision (v1. 27) software environment for measuring TC of the rocks.

**Figure 8:**
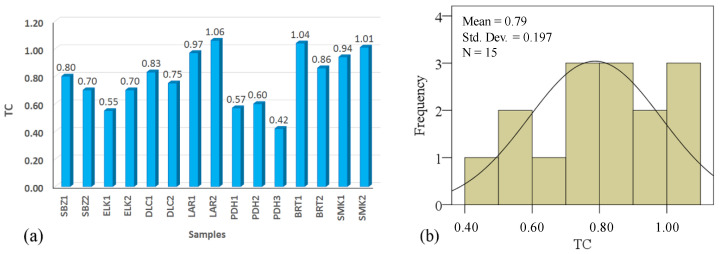
(**a**) Diagram of the texture coefficient (TC) values and (**b**) histogram of the TC for the studied rock samples.

Calculation of TC is possible from TIFF format of microscopic images of rocks using the JMicroVision (v1. 27) program. This is a valuable software program for determining the size, shape, orientation, and texture properties of different rocks. The JMicroVision (v1. 27) environment for calculating TC is presented in Figure 7. The values of TC calculated from Equation (1) for the studied rock samples are presented in Table 4. Additionally, requiremed parameters such as AW, AR, FF, and AF were determined and are summarized in this table. Based on the results, TC values were obtained from “0.42 to 1.06”. The samples of PDH_3_ and LAR_2_ have the lowest and highest values of TC, respectively. A comparative diagram of the values of TC for the studied rocks is shown in Figure 8a. Based on the histogram, the TC has an average value of 0.79, which is presented in Figure 8b. The standard deviation (Std. Dev.) values are very low, which shows a close dispersion of the TC values (Figure 8b). The parameters of AW, AR_1_, and TC have direct relations. Therefore, the value of TC will increase by increasing AW and AR_1_. The results are confirmed by the results presented by Howarth and Rowlands [60], Howarth and Rowlands [61], Ersoy and Waller [62], Gupta and Sharma [63], Tandon and Gupta [64], and Kolay and Baser [69].

## 4. Data Analysis

### 4.1. Simple Regression Analysis (SRA)

**Figure 9:**
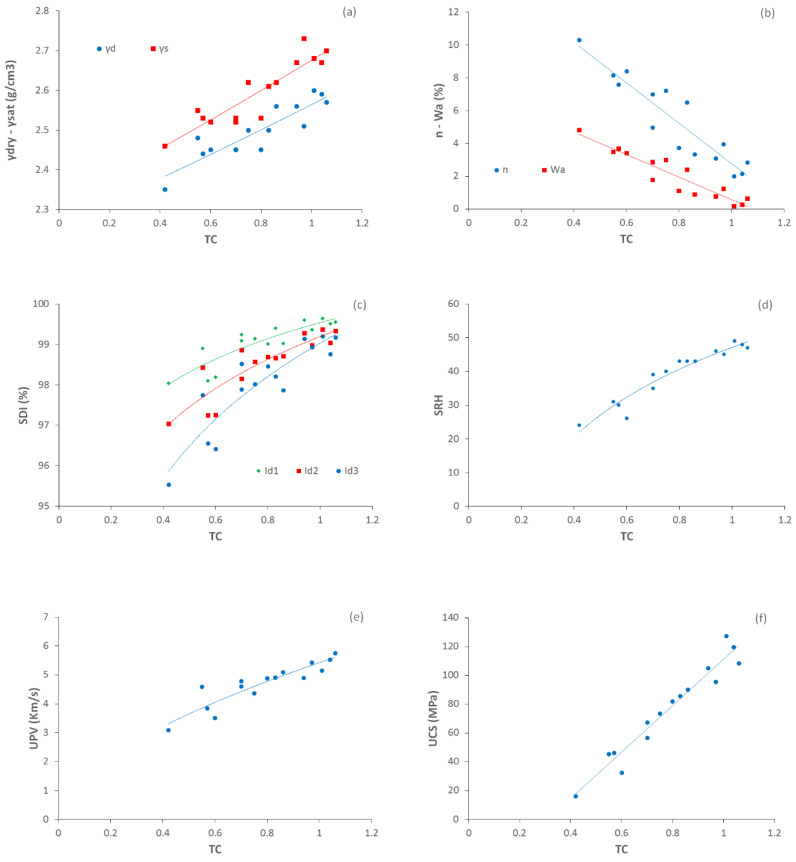
Relations between texture coefficient (TC) and (**a**) dry and saturated unit weights (γ_d_, γ_s_), (**b**) effective porosity (n) and water absorption (W_a_), (**c**) slake durability index (SDI), (**d**) Schmidt rebound hardness (SRH), (**e**) P-wave velocity (UPV), (**f**) uniaxial compressive strength (UCS) for the rocks.

The statistical and regression analyses were conducted by using the IBM SPSS Statistics v. 24 [83] computer program. The index, physical, and mechanical properties including dry and saturated unit weight, effective porosity, water absorption, slake durability index, Schmidt hardness, P-wave velocity, and uniaxial compressive strength were considered as independent variables and the TC was used as the dependent variable. Graphs of these correlations are presented in Figure 9. Additionally, the obtained predictive models and coefficient of determination (R^2^) are presented in Table 5. The R^2^ shows how the values will fit the data. It is calculated by the following equation:

(7) $$R^{2}=1-\frac{\sum_{i=1}^{N}{\left(y-\;y^{\prime }\right)}^{2}}{\sum_{i=1}^{N}{\left(y-\;\tilde{y}\right)}^{2}}$$

**Figure 10:**
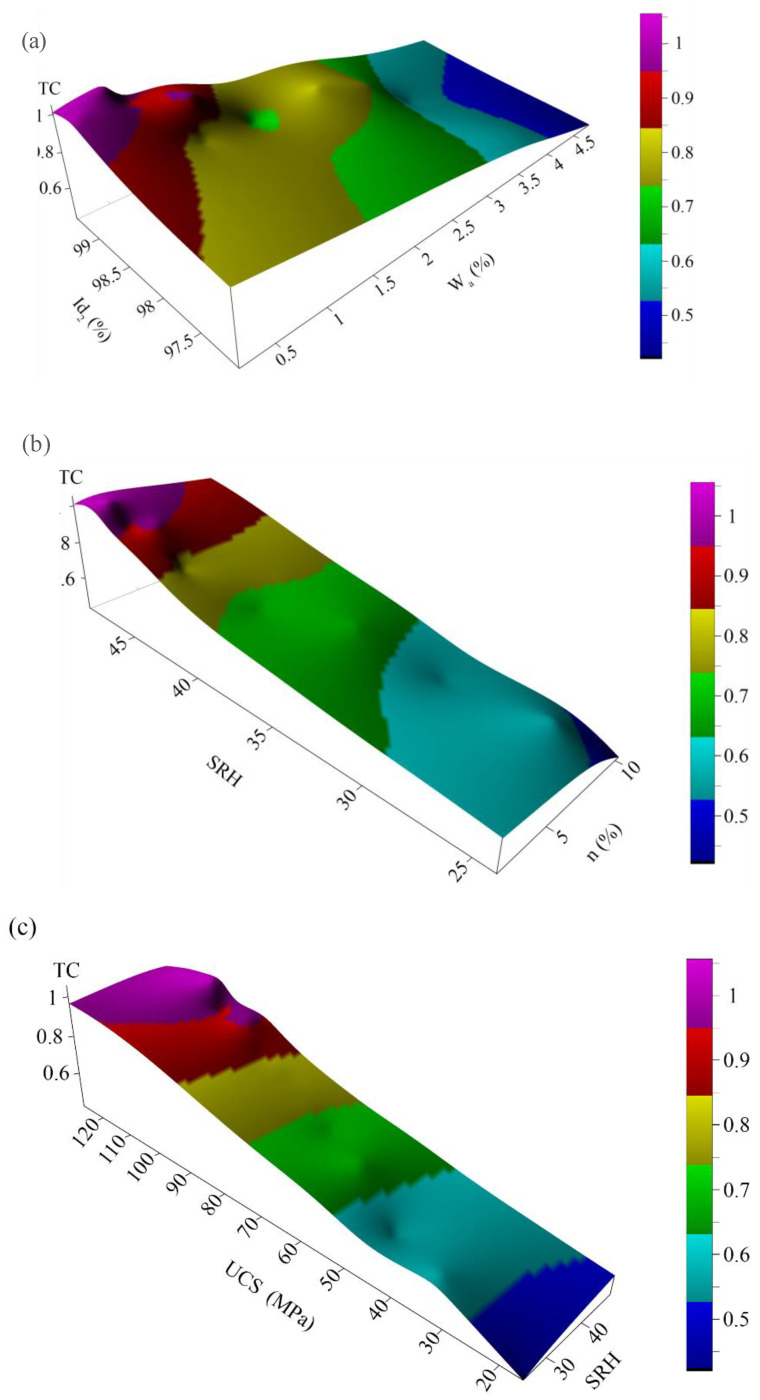
Three-dimensional view of the correlations among (**a**) texture coefficient (TC), slake durability index (Id_2_) and water absorption (W_a_); (**b**) texture coefficient (TC), Schmidt rebound hardness (SRH) and porosity; (**c**) texture coefficient (TC), uniaxial compressive strength (UCS), and Schmidt rebound hardness (SRH).

The results of this evaluation show that R^2^ is between 0.77 and 0.94 (similar to Gupta and Sharma [63] and Khanlari et al. [59]). Figure 8a shows direct exponential relationships between dry and saturated unit weights (γ_d_, γ_s_) and TC. High coefficients of determination (R^2^) of 0.80 and 0.83 were obtained from these evaluations. This means that the density of the studied rock samples is greatly affected by the values of TC. Relationships between porosity and water absorption and TC are inverse linear relations (Figure 9b) with high coefficients of determination (R^2^) of 0.86 and 0.88, respectively. Figure 9c shows relations between slake durability indexes and TC. For this purpose, Id_1_, Id_2_, and Id_3_ were separately correlated to TC. There were direct power relations with good coefficients of determination (R^2^) of 0.77, 0.78, and 0.82, respectively. As can be seen, when the cycles of the slake durability test increase, the coefficient of determination increases. Therefore, correlations between SDI and TC present better results in upper cycles of the test. As can be seen in Figure 9d, a good direct logarithmic relationship is recognizable between SRH and TC with a high coefficient of determination (R^2^) of 0.82. A direct power relation was found between TC and UPV with a coefficient of determination (R^2^) of 0.85, which is shown in Figure 9e. Figure 9 shows a direct linear relation between UCS and TC with a very good coefficient of determination (R^2^) of 0.94. Direct relationships of SDI, SRH, UPV, and UCS with TC indicated that the engineering properties of the rocks are well correlated to texture coefficients (TC). A three-dimensional view of the correlations among texture coefficient (TC), water absorption (W_a_), porosity (n), slake durability index (Id_2_), Schmidt rebound hardness (SRH), and uniaxial compressive strength (UCS) is shown in Figure 10. This figure can simultaneously show the relationship between the TC and two other parameters and expose the simultaneous relationship of these parameters with each other.

To assess the performances of the models, the statistical parameters such as the regression coefficient (R), the root mean square error (RMSE), the coefficient values accounted for (VAF), the mean absolute percentage error (MAPE), and the performance index (PI) were evaluated by the following equations [84]:

(8) $$R=\frac{n\left(\sum^{\;}\mathrm{xy}\right)-\left(\sum^{\;}x\right)\left(\sum^{\;}y\right)}{\sqrt{\left[n\left(\sum^{\;}x^{2}\right)-{\left(\sum^{\;}x\right)}^{2}\right]\left[n\left(\sum^{\;}y^{2}\right)-{\left(\sum^{\;}y\right)}^{2}\right]}}$$

(9) $$\mathrm{RMSE}=\sqrt{\left(\frac{1}{N}\right)}\times \sum_{i=1}^{N}{\left(y-\;y^{\prime }\right)}^{2}$$

(10) $$\mathrm{VAF}=\left[1-\frac{\mathrm{var}y-\;y^{\prime }}{\mathrm{var}y}\right]\times 100$$

(11) $$\mathrm{MAPE}=\left[\frac{1}{N}\sum_{i=1}^{N}\left|\frac{y-\;y^{\prime }}{y}\right|\right]\times 100$$

(12) $$\mathrm{PI}=\left[R^{2}+\left(\frac{\mathrm{VAF}}{100}\right)-\mathrm{RMSE}\right]$$

In these equations, *y* and *y*^′^ are the experimental and calculated values, respectively, and *N* is the total number of points. The models are excellent (the predicted values from the regression equation are equal to the experimental values) if R = 1, RMSE = 0, VAF = 100, MAPE = 0, and PI = 100. Ten predictive models were developed by simple regression analysis (Table 5). The values of parameters related to the model and significance level (Sig.) are presented in Table 6. R values obtained between 0.88 and 0.97 show the high validity of the experimental equations. RMSE values are very low in models 1 and 2, equal to 0.03, that correlate γ_d_ and γ_s_ to the TC. High VAF values were obtained from the models of SRH and UCS. However, mean absolute percentage error values are very low in the SDI models, especially in the Id_2_ model. Performance assessment by PI indicates model 8 has best performance among all models. This model correlates SRH to TC. The significance level for all developed models was calculated equal to 0.000. This indicates that the obtained predictive models by SRA with a 99% confidence limit predict the values of the independent variable when the TC is considered as an input. The results the simple regression analysis revealed that texture coefficients are good parameters for evaluating and predicting the physical, index, and mechanical properties of the studied rock samples.

### 4.2. Multiple Regression Analysis (MRA)

The multiple regression analysis was performed by IBM SPSS Statistics v. 24 [83]. The multiple regression analysis creates a correlation between more than two parameters, and it results in a more accurate correlation with higher values of correlation coefficients (R) and coefficients of determination (R^2^) than simple regression analysis. In the multiple regression analysis, physical properties, namely, dry unit weight and porosity that are determined by a simple and cheap test, and also texture coefficient values were developed in this research and were considered as inputs or independent variables. Other engineering properties including Id_2_, SRH, UPV, and UCS were considered as dependent variables or outputs. Therefore, by three inputs of γ_d_, n, and TC in four models, the values of Id_2_, SRH, UPV, and UCS were predicted.

The summary of coefficients related to multiple regression models, namely, Beta coefficient, standard error of Beta, significance level, lower and upper confidence interval, tolerance, and VIF, is presented in Table 7. The variance inflation factor (VIF) and tolerance are the statistical parameters that show multi-collinearity of models. Based on the results, tolerance values are obtained between 0 and 1. Therefore, the issue of collinearity does not exist in MR models.

Table 8 shows the Rs values such as R, R square, adjusted R, standard error, R Square Change, and Dubrin–Watson for the models. The results in this table show that model 4 has greater R, which estimates UCS with γ_dry_, n, and TC. The Dubrin–Watson (DW) statistic is a number that tests for autocorrelation in the residual form of a statistic regression analysis. The DW statistic is always between 0 and 4. When the value is 0, it indicates negative autocorrelation.

Predictive models that were obtained from the multiple regression analysis and statistical parameters for model performance are presented in Table 9. The predictive model for Id_2_ has lower values of RMSE and MAPE. Model 4, which predicted UCS, has better values of VAF and PI, whereas this model has higher values of RMSE and MAPE. Significance level values indicated that all models predicted output values with a 99% confidence limit. γ_dry_, n, and TC are good parameters to predict the engineering characteristics of the studied rocks based on the regression coefficients of multiple regression analysis. This result is similar to the results of Singh et al. [85], Alber and Kahraman [65], Manouchehrian et al. [42], Ersoy and Acar [66], and Akram et al. [68].

### 4.3. Artificial Neural Network (ANN)

**Figure 11:**
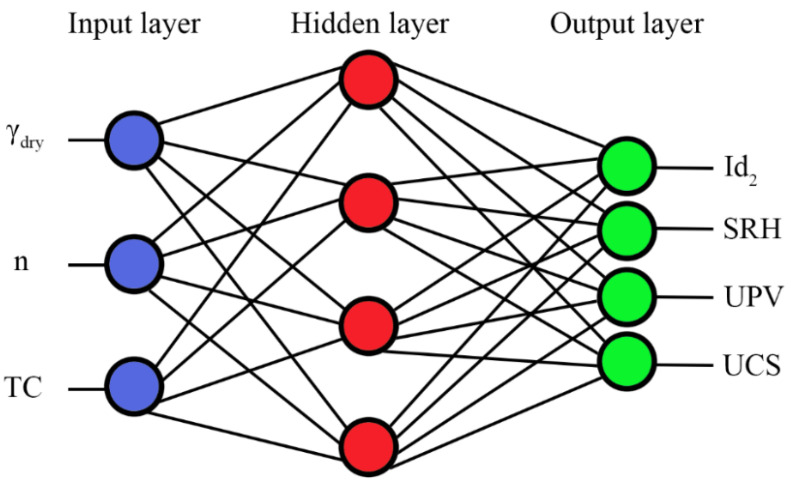
Schematic representation of the constructed neural network.

The artificial neural network is a mathematical and computational approach that is taken from the structural and functional aspects of biological neural networks [86]. It has been widely adopted by scientists due to its ability to develop complex nonlinear models and is applied for solving a wide variety of applications [87]. In the present research, the feed-forward neural network was used to estimate the engineering characteristics of the studied rocks. Similar to the multiple regression models, γ_dry_, n, and TC are constant input parameters in all models. Id_2_, SRH, UPV, and UCS were considered as outputs that were predicted by models 1 to 4, respectively. All data were divided into three data sets for training, validation, and testing. Architectures of ANNs that were used in this research are presented in Figure 11. An ANN toolbox of MATLAB Version 9.1 (R2016b) [88] was used for neural network analyses.

**Figure 12:**
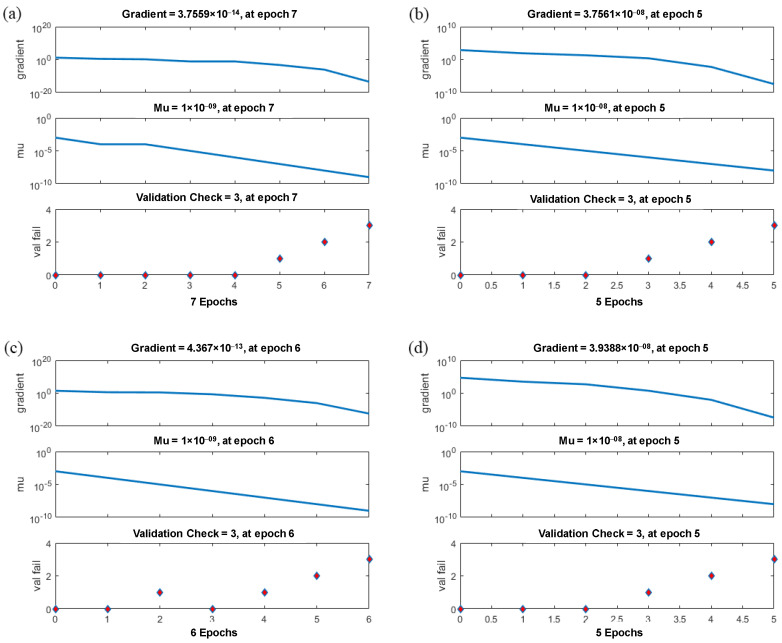
Training state in ANN models for prediction of (**a**) Id_2_, (**b**) SRH, (**c**) UPV, and (**d**) UCS.

**Figure 13:**
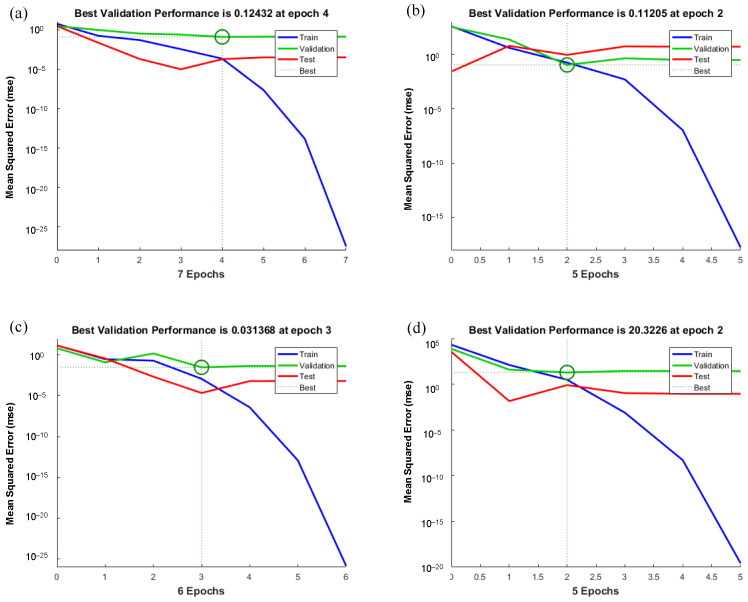
Validation curves in ANN models for prediction of (**a**) Id_2_, (**b**) SRH, (**c**) UPV, and (**d**) UCS.

**Figure 14:**
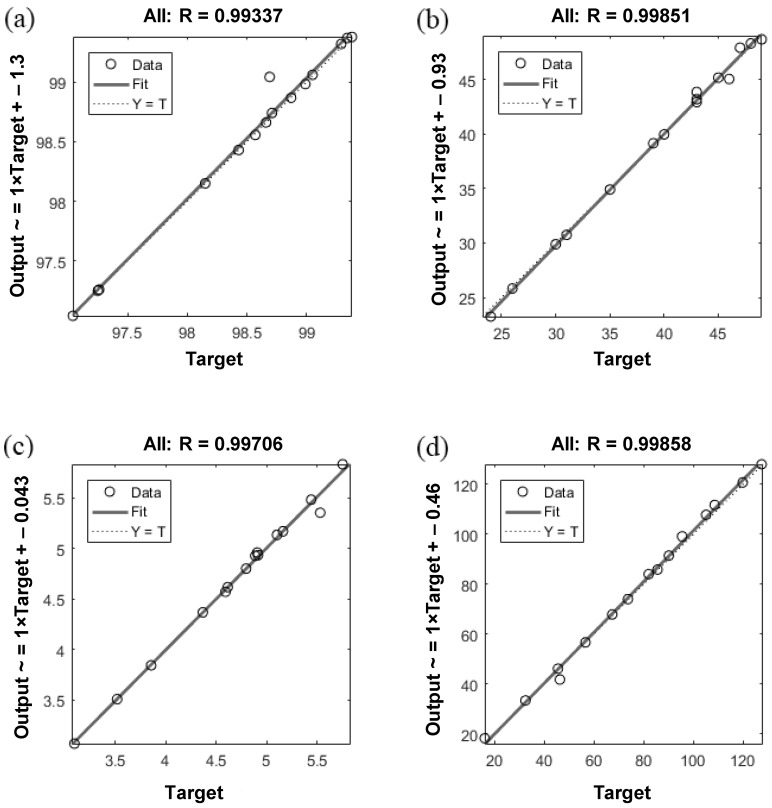
Regression plots of ANN models for predicting (**a**) Id_2_, (**b**) SRH, (**c**) UPV, and (**d**) UCS.

Figure 12 shows the training state for the ANN models for predicting the outputs containing Id_2_, SRH, UPV, and UCS established in the MATLAB environment. As can be seen from this figure, in models 1 to 4 after the epoch numbers of 4, 2, 3, and 2, the errors occur three times, and the test is stopped at different epoch numbers. Since the errors happen three times before stopping the test, the validation checks would be equal to three. Figure 13 shows the validation curves in ANN models for predicting the outputs containing Id_2_, SRH, UPV, and UCS, which are established in the MATLAB (v 9.1) environment. Based on Figure 13a, which shows the predicted Id_2_, the best validation performance is happening at epoch 4, and the test is stopped at epoch 7 after three error repetitions. In models 2 and 4, for predicting SRH and UCS (Figure 13b,d), the test is stopped at epoch 2 after three error repetitions. Figure 13c shows the best validation performance is happening at epoch 3 (in model 3), and the test is stopped at epoch 6 after three error repetitions. The values of mean squared error (MSE), which is found on the *y*-axis in Figure 13, are approximately similar in four models, and in model 1 is less than other models. The plots of regression for training, testing, and validation of the proposed ANN model were established in the MATLAB (v. 9.1) environment. Very accurate correlation coefficients were obtained, as illustrated in Figure 14. In all models, R higher than 0.99 shows the high-performance validity of established models by ANN.

To assess the performance of the results of the ANN models, the statistical parameter was calculated and is presented in Table 10. As can be seen, R values in all models are equal to 0.99. RMSE values, except in model 4 which predicted UCS, are very low, and in the predictive model of UPV is equal to 0.06. The performance index of the UPV model is equal to 99.94, which is high. Despite high values of RMSE and MAPE in model 4, the VAF value is higher than other models. The significance level in all models is equal to 0.000, indicating that predictive models by ANN estimated the output parameters with a 99% confidence limit.

## 5. Discussion

**Figure 15:**
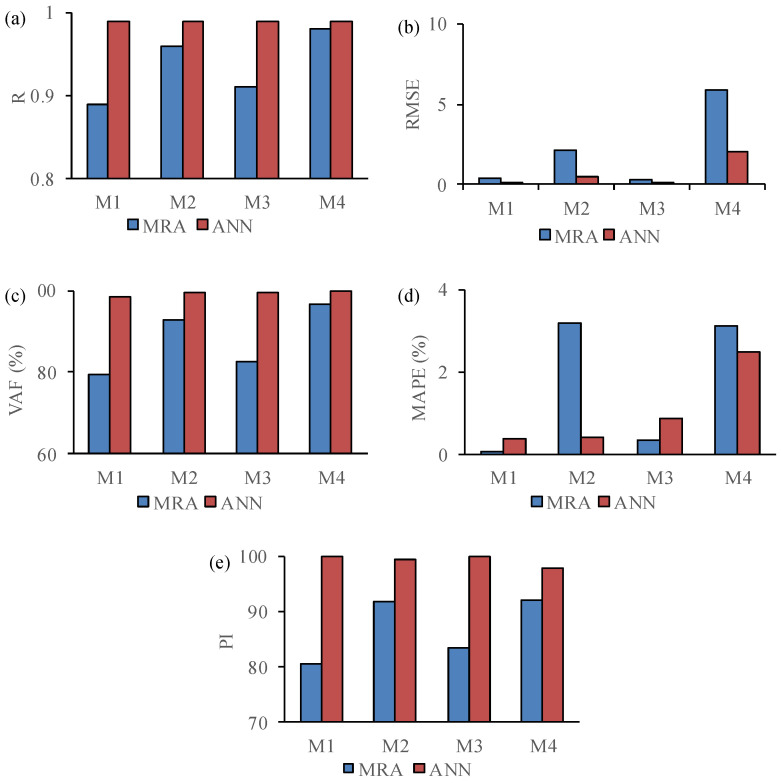
Comparative diagrams of statistical coefficients for results of MRA and ANN models (**a**) R, (**b**) RMSE, (**c**) VAF, (**d**) MAPE, and (**e**) PI.

Statistical parameters, namely R, RMSE, VAF, MAPE, and PI, were evaluated for the developed models by SRA, MRA, and ANN to assess their accuracy. High values of the determination coefficient obtained from the SRA method indicate TC is an able coefficient for engineering properties estimation of the studied rocks. In MRA and ANN models, γ_dry_, n, and TC were considered as inputs and four models were created for estimating Id_2_, SRH, UPV, and UCS of the studied samples. Then, the statistical parameter assesses the results of the MR and ANN models. A comparative diagram of R, RMSE, VAF, MAPE, and PI for the results of MRA and ANN models is presented in Figure 15. It is clear that the ANN models have better performance than the MR models. Except in model 4, for predicting UCS that shows approximately similar results for MRA and ANN models, the other models have valuable differences in statistical parameters. A higher R value for the ANN models means that there is a better relationship between experimental and calculated values in comparison to the MRA model. This result is confirmed by the research of Alber and Kahraman [65], Manouchehrian et al. [42], and Ersoy and Acar [66].

Table 11 presents the experimental and calculated values of Id_2_, SRH, UPV, and UCS provided by the laboratory tests and models of MR and ANN together with the obtained standard deviation (S.D.) for all models. The results show that the values of the mean and standard deviation in ANN models are approximately equal to experimental values, which indicates less variety than MR models.

**Figure 16:**
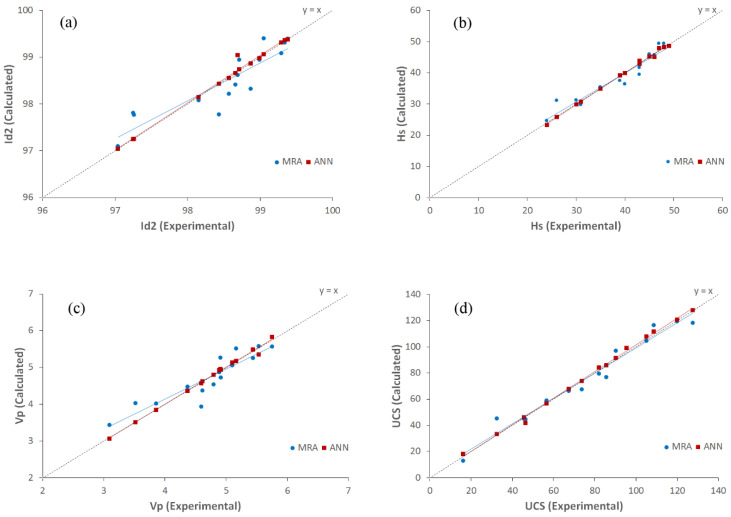
Plots of experimental and calculated values of (**a**) Id_2_, (**b**) SRH, (**c**) UPV, and (**d**) UCS from the MRA and ANN.

The experimental and calculated values of Id_2_, SRH, UPV, and UCS have been plotted in diagrams of Figure 15 to compare the results of MRA and ANN for predicting the engineering characteristics of the studied rocks. The adaption rate of trend lines of MRA and ANN with the line y = x show the validity of the models. The correlations between experimental and calculated values of Id_2_ that were obtained from γ_dry_, n, and TC are shown in Figure 16a. Based on these graphs, the trend line of ANN completely fits the line y = x, whereas the MRA trend line approximately intercepts the line y = x. This shows the moderate validity of the equations of the MRA for estimating Id_2_. The correlations between experimental and calculated values of SRH are shown in Figure 16b. As can be seen, the MRA and ANN trend lines approximately overlap together and show that both methods in this model obtained the same results for predicting Schmidt rebound hardness. Figure 16c shows the relationships between experimental results and calculated values of UPV. It is obvious that ANN estimated values of UPV are closer to the corresponding calculated values than MRA models. The trend line of the MRA model does not completely fit the 45° line and the results of the ANN are better than those. The correlations between experimental and calculated values of UCS are shown in Figure 16d. It shows that MRA and ANN trend lines of these diagrams entirely overlap together. So, both methods in this model have the same results for estimating the uniaxial compressive strength. Therefore, based on the results of different models, the predicted values from ANN models highly fit 45° lines and show the high validity of this method.

**Figure 17:**
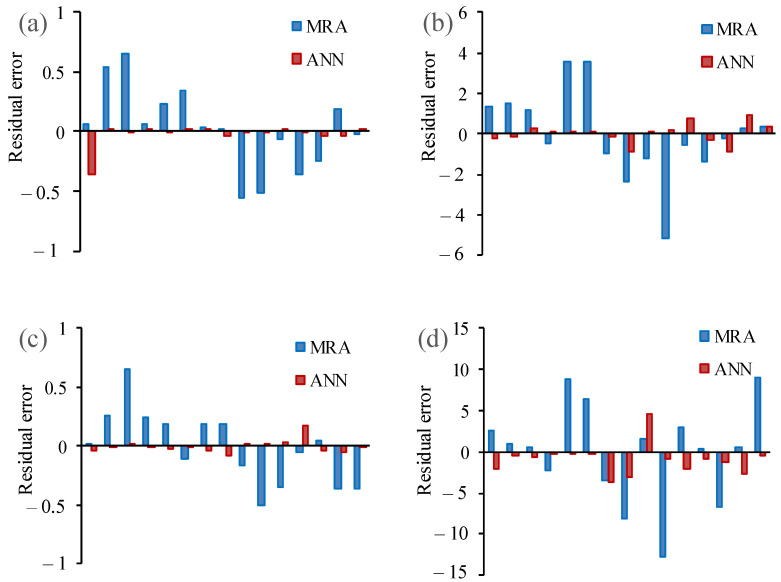
Residual error plots of MRA and ANN models to predict (**a**) Id_2_, (**b**) SRH, (**c**) UPV, and (**d**) UCS.

For the final assessment, residual error values were calculated for the results of MR and ANN models (Table 12). These values were calculated by subtraction of experimental and calculated values based on MRA and ANN created models. To compare the obtained values of residual error, their graphs were depicted for Id_2_, SRH, UPV, and UCS models, respectively (Figure 17). Very low values of S.D. of ANN models show the validity of this method for predicting engineering characteristics of the studied rocks. Additionally, it was found that the average values of residual error in ANN models had a tendency to be 0.00, for instance, in model 3. Based on Figure 17, the ANN model has a lower residual error than the MR model. It can be seen that the error trend lines of ANN are more fit to the horizontal line (*x*-axis).

## 6. Conclusions

In this research, various limestone and sandstone rock samples were investigated to develop predictive models between their engineering properties including dry unit weight (γ_dry_), saturated unit weight (γ_sat_), water absorption (W_a_), porosity (n), Schmidt rebound hardness (SRH), ultrasonic P-wave velocity (UPV), and uniaxial compressive strength (UCS). The texture investigations carried out by using JMicroVision (v1. 27) software led to texture coefficients (TC). Predictive models were developed by statistical and soft computing methods including simple regression analysis (SRA), multiple regression analysis (MRA), and artificial neural network (ANN). Ten experimental equations were developed by the SRA for predicting γ_d_, γ_s_, n, W_a_, SDI, SRH, UPV, and UCS from the TC values with very high values of Pearson regression coefficient (R). It was found that the TC has direct relations with γ_d_, γ_s_, SDI, SRH, UPV, and UCS, and inverse relations with n and W_a_. Additionally, four predictive models were developed by the MRA and ANN methods where γ_d_, n, and TC were considered as inputs and Id_2_, SRH, UPV, and UCS were considered as outputs. The values of the obtained correlation coefficients were very high from the MRA. In the ANN, the feed-forward neural network was used to establish predictive models, and the R value was obtained equal to 0.99 in all models. Statistical parameters including the RMSE, VAF, MAPE, and PI were evaluated and compared to each other for assessing the performance of the MRA and ANN models. The calculated statistical parameters indicated that the ANN models have better performance than the MR models. The experimental and calculated values of Id_2_, SRH, UPV, and UCS obtained from the laboratory tests and predicted by the MRA and ANN models were approximately equal to each other. Additionally, the results of residual error analysis show lower standard deviations for predicted data by the ANN models than the MRA ones. The results of this research indicated texture coefficient analysis is a reliable method (as mentioned in Ersoy and Waller [62], Singh et al. [36], Alber and Kahraman [65], Gupta and Sharma [63], Manouchehrian et al. [42], Tandon and Gupta [64], Akram et al. [68], Kolay and Baser [69]) to predict engineering properties of the studied rocks. Therefore, by using the obtained results from the research and statistical methods as well as artificial neural networks, it is successfully passible to establish credible models for predicting engineering properties of other rocks. This can be an advantage of the present research. On the other hand, indirect estimation of engineering properties of rocks is not always as accurate as direct laboratory methods and this can be a limitation for this research and similar research.

## Figures and Tables

**Table 1:** Mineralogical and petrographic properties of the studied rock samples.

Sample	Lithology	Minerals (%)					
		Qtz.	Cal.	Dol.	Fld.	Fos.	Other Minerals
SBZ_1_	Dolomitic limestone	10	60	25	-	-	5
SBZ_2_	Dolomitic limestone	20	40	35	-	-	5
ELK_1_	Ooid grainstone (limestone)	5	10	-	-	80	5
ELK_2_	Ooid grainstone (limestone)	5	20	-	-	70	5
DLC_1_	Sandy grainstone (limestone)	20	65	5	-	-	10
DLC_2_	Sandy grainstone (limestone)	25	60	5	-	-	10
LAR_1_	Microcrystalline limestone	15	70	-	-	12	3
LAR_2_	Microcrystalline limestone	20	63	-	-	15	2
PDH_1_	Greywacke (sandstone)	70	15	10	-	-	5
PDH_2_	Greywacke (sandstone)	55	30	10	-	-	5
PDH_3_	Greywacke (sandstone)	55	30	10	-	-	5
BRT_1_	Litharenite (sandstone)	70	10	-	15	-	5
BRT_2_	Litharenite (sandstone)	60	15	-	20	-	5
SMK_1_	Sub litharenite (sandstone)	70	7	-	10	-	13
SMK_2_	Sub litharenite (sandstone)	80	7	-	5	-	8

Note: Qtz.: Quartz (SiO_2_); Cal.: Calcite (CaCO_3_); Dol.: Dolomite (Ca.Mg(CO_3_)_2_); Fld.: Feldspar; Fos.: Fossil.

**Table 2:** Values of engineering properties of the tested rock samples.

Sample	γ_dry_ (g/cm^3^)	γ_sat_ (g/cm^3^)	n (%)	W_a_ (%)	Id_1_ (%)	Id_2_ (%)	Id_3_ (%)	SRH	UPV (Km/s)	UCS (MPa)
SBZ_1_	2.45	2.53	3.74	1.12	99.02	98.69	98.46	43	4.89	81.99
SBZ_2_	2.45	2.53	4.98	1.78	99.25	98.87	98.52	39	4.80	67.40
ELK_1_	2.48	2.55	8.15	3.49	98.91	98.43	97.75	31	4.59	45.54
ELK_2_	2.45	2.52	7.01	2.87	99.10	98.15	97.89	35	4.61	56.58
DLC_1_	2.50	2.61	6.50	2.40	99.41	98.66	98.21	43	4.91	85.59
DLC_2_	2.50	2.62	7.21	2.98	99.15	98.57	98.02	40	4.37	73.67
LAR_1_	2.51	2.73	3.96	1.24	99.37	98.99	98.94	45	5.44	95.45
LAR_2_	2.57	2.70	2.85	0.64	99.56	99.34	99.18	47	5.75	108.55
PDH_1_	2.44	2.53	7.58	3.69	98.10	97.25	96.56	30	3.85	46.29
PDH_2_	2.45	2.52	8.41	3.42	98.19	97.26	96.42	26	3.52	32.46
PDH_3_	2.35	2.46	10.32	4.82	98.04	97.04	95.54	24	3.09	16.09
BRT_1_	2.59	2.67	2.15	0.26	99.52	99.05	98.76	48	5.53	119.82
BRT_2_	2.56	2.62	3.33	0.90	99.03	98.71	97.87	43	5.10	90.12
SMK_1_	2.56	2.67	3.10	0.77	99.61	99.29	99.15	46	4.91	105.02
SMK_2_	2.60	2.68	2.00	0.18	99.65	99.38	99.21	49	5.16	127.43

**Table 3:** Descriptive results of linear regression analysis for correlating the engineering properties of the rocks.

Correlations											
		γ_dry_	γ_sat_	n	W_a_	Id_1_	Id_2_	Id_3_	SRH	UPV	UCS
G_s_	R										
	Sig.										
γ_dry_	R	1									
	Sig.										
γ_sat_	R	0.875	1								
	Sig.	0.000									
n	R	−0.845	−0.768	1							
	Sig.	0.000	0.001								
W_a_	R	−0.855	−0.774	0.994	1						
	Sig.	0.000	0.001	0.000							
Id_1_	R	0.796	0.784	−0.803	−0.833	1					
	Sig.	0.000	0.001	0.000	0.000						
Id_2_	R	0.820	0.801	−0.869	−0.889	0.971	1				
	Sig.	0.000	0.000	0.000	0.000	0.000					
Id_3_	R	0.792	0.794	−0.868	−0.884	0.964	0.978	1			
	Sig.	0.000	0.000	0.000	0.000	0.000	0.000				
SRH	R	0.849	0.843	−0.930	−0.937	0.923	0.940	0.932	1		
	Sig.	0.000	0.000	0.000	0.000	0.000	0.000	0.000			
UPV	R	0.813	0.810	−0.878	−0.887	0.900	0.924	0.929	0.917	1	
	Sig.	0.000	0.000	0.000	0.000	0.000	0.000	0.000	0.000		
UCS	R	0.914	0.873	−0.945	−0.948	0.891	0.909	0.902	0.980	0.891	1
	Sig.	0.000	0.000	0.000	0.000	0.000	0.000	0.000	0.000	0.000	

Note: R: Pearson correlation coefficient and Sig.: significant level, Sig. (two-tailed).

**Table 4:** Texture coefficient derivations for the rocks.

Sample	AW	$\frac{N_{0}}{N_{0}+N_{1}}$	$\frac{1}{{\mathrm{FF}}_{0}}$	$\frac{N_{0}}{N_{0}+N_{1}}$	AR_1_	AF_1_	TC
SBZ_1_	0.66	0.81	1.29	0.19	1.73	0.5	0.8
SBZ_2_	0.66	0.82	1.13	0.18	1.65	0.5	0.7
ELK_1_	0.47	0.92	1.19	0.08	1.56	0.5	0.55
ELK_2_	0.6	0.84	1.21	0.16	1.74	0.5	0.7
DLC_1_	0.8	0.92	1.06	0.08	1.55	0.5	0.83
DLC_2_	0.71	0.9	1.1	0.1	1.53	0.5	0.75
LAR_1_	0.86	0.81	1.18	0.19	1.75	0.5	0.97
LAR_2_	0.98	0.83	1.14	0.17	1.69	0.5	1.06
PDH_1_	0.57	0.91	1.03	0.09	1.46	0.5	0.57
PDH_2_	0.58	0.97	1.04	0.03	1.47	0.5	0.6
PDH_3_	0.38	0.96	1.1	0.04	1.52	0.5	0.42
BRT_1_	0.87	0.83	1.25	0.17	1.8	0.5	1.04
BRT_2_	0.77	0.83	1.16	0.17	1.85	0.5	0.86
SMK_1_	0.85	0.85	1.15	0.15	1.68	0.5	0.94
SMK_2_	0.9	0.82	1.17	0.18	1.79	0.5	1.01

Note: The meaning of the acronyms and letters in the table were previously presented.

**Table 5:** Simple regression models for texture coefficient (TC) of the studied rocks.

Model	Parameter	Predictive Model	Equation Type	R^2^
1	TC—γ_d_	γ_d_ = 2.261 × e^0.1259TC^	Exponential	R^2^ = 0.80
2	TC—γ_s_	γ_s_ = 2.3149 × e^0.1451TC^	Exponential	R² = 0.83
3	TC—n	n = −12.328TC + 15.118	Linear	R² = 0.86
4	TC—W_a_	W_a_ = −6.8834TC + 7.4529	Linear	R² = 0.88
5	TC—Id_1_	Id_1_ = 99.541TC^0.0178^	Power	R² = 0.77
6	TC—Id_2_	Id_2_ = 99.196TC^0.0255^	Power	R² = 0.78
7	TC—Id_3_	Id_3_ = 99.033TC^0.0375^	Power	R² = 0.82
8	TC—H_S_	SRH = 28.948ln(TC) + 47.149	Logarithmic	R² = 0.92
9	TC—V_P_	UPV = 5.423TC^0.5718^	Power	R² = 0.82
10	TC—UCS	UCS = 161.08TC − 49.913	Linear	R² = 0.94

**Table 6:** Statistical parameters for performance of SRA models.

Model	R	RMSE	VAF (%)	MAPE (%)	PI	Sig. (Two-Tailed)
1	0.89	0.03	79.73	2.06	79.77	0.000
2	0.91	0.03	82.47	2.76	83.79	0.000
3	0.93	0.95	85.92	40.52	85.91	0.000
4	0.94	0.48	87.98	74.08	88.40	0.000
5	0.88	0.25	75.85	0.13	77.51	0.000
6	0.88	0.35	78.27	0.06	78.44	0.000
7	0.91	0.45	81.70	0.26	83.36	0.000
8	0.96	2.17	92.42	5.37	90.75	0.000
9	0.91	0.30	82.20	2.30	83.52	0.000
10	0.97	7.56	94.26	3.71	87.38	0.000

**Table 7:** Multiple regression coefficients for the developed models.

Variable										
Model		Unstandardized Coefficients		Standardized Coefficients	t	Sig.	95.0% Confidence Interval for B		Collinearity Statistics	
		B	Std. Error	Beta			Lower Bound	Upper Bound	Tolerance	VIF
1	Id_2_	93.635	7.982		11.731	0.000	76.068	111.203		
	γ_d_	1.743	3.392	0.157	0.514	0.618	−5.723	9.209	0.200	4.991
	n	−0.115	0.107	−0.391	−1.068	0.308	−0.351	0.122	0.139	7.183
	TC	1.457	1.694	0.373	0.860	0.408	−2.272	5.185	0.099	10.114
2	SRH	35.307	50.731		0.696	0.501	−76.351	146.965		
	γ_d_	−5.283	21.559	−0.045	−0.245	0.811	−52.735	42.169	0.200	4.991
	n	−1.013	0.682	−0.325	−1.485	0.166	−2.515	0.488	0.139	7.183
	TC	28.785	10.767	0.694	2.673	0.022	5.087	52.483	0.099	10.114
3	UPV	2.969	7.110		0.418	0.684	−12.681	18.619		
	γ_d_	0.131	3.022	0.012	0.043	0.966	−6.520	6.782	0.200	4.991
	n	−0.082	0.096	−0.288	−0.853	0.412	−0.292	0.129	0.139	7.183
	TC	2.349	1.509	0.624	1.556	0.148	−0.973	5.670	0.099	10.114
4	UCS	−215.929	138.709		−1.557	0.148	−521.225	89.367		
	γ_d_	98.474	58.948	0.208	1.671	0.123	−31.269	228.217	0.200	4.991
	n	−3.680	1.865	−0.295	−1.973	0.074	−7.786	0.425	0.139	7.183
	TC	84.860	29.440	0.511	2.883	0.015	20.064	149.656	0.099	10.114

Note: γ_d_, n, and TC are independent variables (predictors); Id_2_, SRH, UPV, and UCS are dependent variables.

**Table 8:** Various Rs values for the selected models.

Model Summary ^a^										
Model	R	R Square	Adjusted R Square	Std. Error of the Estimate	Change Statistics					Durbin–Watson
					R Square Change	F Change	df1	df2	Sig. F Change	
1	0.892 ^b^	0.795	0.739	0.39213	0.795	14.220	3	11	0.000	0.919
2	0.963 ^c^	0.927	0.907	2.49232	0.927	46.397	3	11	0.000	1.206
3	0.908 ^d^	0.825	0.778	0.34932	0.825	17.315	3	11	0.000	0.814
4	0.983 ^e^	0.966	0.956	6.81453	0.966	103.421	3	11	0.000	1.931

a. Predictors (constants): TC, γ_d_, n; b. Dependent variable: Id_2_; c. Dependent variable: SRH; d. Dependent variable: UPV; e. Dependent variable: UCS.

**Table 9:** Predictive models from multiple regression analysis and statistical parameters for performance of the models.

Model	Predictive Model	RMSE	VAF (%)	MAPE (%)	PI	Sig. (Two-Tailed)
1	Id_2_ = 93.64 + 1.74γ_d_ − 0.12n + 1.46TC	0.34	79.47	0.07	80.46	0.000
2	SRH = 35.31 − 5.28γ_d_ − 1.01n + 28.79TC	2.13	92.68	3.19	91.79	0.000
3	UPV = 2.97 + 0.13γ_d_ − 0.08n + 2.35TC	0.30	82.52	0.34	83.53	0.000
4	UCS = −215.93 + 98.47γ_d_ − 3.68n + 84.86TC	5.84	96.58	3.10	92.13	0.000

**Table 10:** Statistical parameters for performance of ANN models.

Model	R	RMSE	VAF (%)	MAPE (%)	PI	Sig. (Two-Tailed)
1	0.99	0.09	98.60	0.36	99.89	0.000
2	0.99	0.48	99.63	0.42	99.22	0.000
3	0.99	0.06	99.39	0.85	99.94	0.000
4	0.99	2.05	99.67	2.47	99.67	0.000

**Table 11:** Experimental and calculated values of Id_2_, SRH, UPV, and UCS obtained from the MRA and ANN models.

Sample	Model 1 (Id_2_)			Model 2 (SRH)			Model 3 (UPV)			Model 4 (UCS)		
	Ex.	MRA	ANN	Ex.	MRA	ANN	Ex.	MRA	ANN	Ex.	MRA	ANN
SBZ_1_	98.69	98.62	99.04	43	41.63	43.18	4.89	4.87	4.93	81.99	79.45	84.02
SBZ_2_	98.87	98.33	98.87	39	37.50	39.14	4.80	4.54	4.80	67.40	66.4	67.88
ELK_1_	98.43	97.78	98.43	31	29.82	30.74	4.59	3.93	4.57	45.54	44.96	46.14
ELK_2_	98.15	98.08	98.15	35	35.45	34.89	4.61	4.37	4.62	56.58	58.93	56.67
DLC_1_	98.66	98.42	98.66	43	39.44	42.91	4.91	4.73	4.93	85.59	76.76	85.86
DLC_2_	98.57	98.22	98.56	40	36.42	39.95	4.37	4.48	4.37	73.67	67.36	74.01
LAR_1_	98.99	98.95	98.98	45	45.98	45.15	5.44	5.26	5.48	95.45	98.97	99.09
LAR_2_	99.34	99.32	99.37	47	49.38	47.90	5.75	5.57	5.83	108.55	116.6	111.65
PDH_1_	97.25	97.81	97.25	30	31.18	29.88	3.85	4.02	3.84	46.29	44.81	41.78
PDH_2_	97.26	97.77	97.26	26	31.15	25.82	3.52	4.03	3.51	32.46	45.29	33.38
PDH_3_	97.04	97.10	97.04	24	24.57	23.25	3.09	3.44	3.07	16.09	13.14	18.22
BRT_1_	99.05	99.41	99.06	48	49.40	48.30	5.53	5.58	5.35	119.82	119.45	120.68
BRT_2_	98.71	98.95	98.74	43	43.19	43.84	5.10	5.06	5.13	90.12	96.88	91.46
SMK_1_	99.29	99.09	99.32	46	45.72	45.03	4.91	5.26	4.96	105.09	104.51	107.73
SMK_2_	99.38	99.40	99.38	49	48.64	48.67	5.16	5.52	5.17	127.43	118.44	127.97
Mean	98.51	98.48	98.54	39.27	39.30	39.24	4.70	4.71	4.70	76.80	76.80	77.77
S.D.	0.77	0.70	0.77	8.16	7.85	8.36	0.74	0.67	0.74	32.64	32.08	33.30

**Table 12:** Residual error values of MR and ANN models.

Sample	Model 1 (Id_2_)		Model 2 (SRH)		Model 3 (UPV)		Model 4 (UCS)	
	MRA	ANN	MRA	ANN	MRA	ANN	MRA	ANN
SBZ_1_	0.068	−0.353	1.371	−0.183	0.017	−0.042	2.544	−2.027
SBZ_2_	0.543	0.003	1.503	−0.141	0.261	−0.005	1.003	−0.479
ELK_1_	0.650	0.000	1.181	0.264	0.659	0.021	0.583	−0.599
ELK_2_	0.066	0.000	−0.447	0.107	0.240	−0.005	−2.347	−0.087
DLC_1_	0.238	−0.001	3.559	0.089	0.188	−0.021	8.831	−0.269
DLC_2_	0.350	0.014	3.580	0.049	−0.115	−0.001	6.313	−0.339
LAR_1_	0.042	0.007	−0.984	−0.155	0.182	−0.042	−3.521	−3.639
LAR_2_	0.023	−0.029	−2.379	−0.900	0.186	−0.080	−8.051	−3.104
PDH_1_	−0.558	0.000	−1.181	0.123	−0.166	0.012	1.477	4.508
PDH_2_	−0.510	0.000	−5.154	0.176	−0.509	0.010	−12.829	−0.919
PDH_3_	−0.064	0.000	−0.571	0.752	−0.344	0.027	2.952	−2.133
BRT_1_	−0.357	−0.011	−1.405	−0.295	−0.049	0.177	0.370	−0.857
BRT_2_	−0.240	−0.030	−0.189	−0.840	0.045	−0.032	−6.758	−1.339
SMK_1_	0.195	−0.029	0.275	0.970	−0.358	−0.054	0.576	−2.645
SMK_2_	−0.019	0.002	0.360	0.335	−0.361	−0.008	8.989	−0.536
Mean	0.03	−0.03	−0.03	0.02	−0.01	0.00	0.01	−0.96
S.D.	0.34	0.09	2.13	0.48	0.30	0.06	5.84	1.81

## Data Availability

Not applicable.
